# Serum Vitamin D Level in Patients with Coronary Artery Disease and Association with Sun Exposure: Experience from a Tertiary Care, Teaching Hospital in India

**DOI:** 10.1155/2019/6823417

**Published:** 2019-02-03

**Authors:** Tauseef Akhtar, Ramesh Aggarwal, Sachin Kumar Jain

**Affiliations:** ^1^Department of Medicine, Lady Hardinge Medical College and Dr. Ram Manohar Lohia Hospital, New Delhi, India; ^2^Department of Medicine, John H. Stroger Jr. Hospital of Cook County, Chicago, Illinois, USA

## Abstract

**Background:**

Vitamin D, a fat-soluble vitamin, has various extraskeletal effects, and several human and animal studies have suggested that vitamin D deficiency may be a contributory factor in the pathogenesis of coronary artery disease (CAD). However, such studies in the Indian subcontinent are either lacking or have shown conflicting results.

**Methods:**

This was a descriptive cross-sectional study involving 121 patients with CAD from a tertiary care center and their 80 age-matched healthy controls. Serum vitamin D levels along with serum and urine chemistries were measured in both the groups. The average duration of sun exposure/day and use of sunscreen were also considered in the study cohort using a questionnaire. Serum vitamin D levels were categorized into deficient (<30 nmol/lit), insufficient (30–75 nmol/lit), and sufficient (>75 nmol/lit) groups.

**Results:**

Among the cases, 51.2% of the patients were vitamin D deficient and 44.6% patients had insufficient vitamin D levels, whereas among controls, 40% and 31% of the population had deficient and insufficient levels of vitamin D, respectively. However, the mean value of the serum vitamin D level was not statistically different in the cases as compared to that of the controls (34.06 vs 40.19 nmol/lit) (*P*=0.08). Corrected serum calcium (9.26 vs 9.59 mg%) (*P* ≤ 0.0001) and serum albumin levels (4.21 vs 4.75 gm%) (*P* ≤ 0.0001) were lower in the cases than those of the controls. The average sun exposure/day was higher among the cases than that among the controls (2.93 vs 1.85 hours) (*P*=0.001).

**Conclusion:**

Vitamin D deficiency is widely prevalent in Indian population despite abundant sunshine, and the duration of sun exposure is not correlated with serum vitamin D levels. Vitamin D deficiency is not associated with CAD. However, serum calcium is deficient in CAD patients as compared to the controls. Large-scale studies are required to explore the association further to evaluate the benefits of screening and correction of vitamin D deficiency in patients with CAD.

## 1. Introduction

Vitamin D, a fat-soluble vitamin also known as an antiricketic factor or sunshine vitamin, is unique in the sense that the body synthesizes it, and it also functions as a hormone. Besides its pivotal role in calcium homeostasis and bone mineral metabolism, the vitamin D endocrine system is now recognized to be involved in a wide range of fundamental biological functions in cell differentiation, inhibition of cell growth, and immunomodulation. Several human and animal studies have suggested that vitamin D deficiency may be a contributory factor in the pathogenesis of coronary artery disease (CAD). Vitamin D deficiency has been shown to result in cardiac hypertrophy and fibrosis by elevation of matrix metalloproteinase enzyme [1, 2]. It also predisposes to hypertension by upregulation of the renin-angiotensin system [3]. Similarly, it is also involved in promoting the formation of atheromatous plaque by increasing the uptake of lipids by macrophages and their conversion into foam cells [4]. CAD is an emerging problem in developing countries like India and responsible for significant morbidity and mortality, and the situation is expected to be worse in the future [5]. Multiple studies have shown that vitamin D deficiency is widely prevalent in the Indian subcontinent [6–9], and also, there are studies reporting an early onset and aggressive CAD in South Asian population [10, 11], However, only a few studies explore the role of vitamin D deficiency in CAD patients from the Indian subcontinent and have shown conflicting results [12–16]. This study was undertaken to examine the association of vitamin D deficiency in patients with CAD and to assess the serum vitamin D level in relation to the duration of sun exposure.

## 2. Materials and Methods

This was a descriptive cross-sectional study conducted at a tertiary care center and teaching hospital in New Delhi. The protocol for the study was duly approved by the ethical committee of our institute. Participation in the study was voluntary, and informed consent was obtained from all the participants of the study before enrollment. About 121 cases who were admitted to our hospital or who attended a cardiology clinic and their 80 age-matched healthy controls from the year 2010 to 2012 were enrolled. Subjects were included if they had CAD proved by either angiography or ECG changes and suggestive symptoms with elevated biomarkers (CK-MB and troponin) belonging to the age group of 40 to 70 years. Subjects were excluded from the study if they had diabetes mellitus, liver insufficiency, acute and chronic kidney diseases, history of intake of vitamin D supplements, and consumption of drugs like rifampicin, phenytoin, barbiturate, and thiazide diuretics which interfere with vitamin D metabolism. Subjects with tuberculosis and a history suggestive of chronic malabsorption were also excluded from the study.

Study participants were administered a questionnaire to determine the symptoms of vitamin D deficiency, average duration of sun exposure/day, and use of sunscreen. Anthropometric measurements were taken, and blood samples were drawn in vacutainer tubes under sterile conditions. Samples were allowed to clot at room temperature and then centrifuged for 5 minutes. Serum was then collected and stored at −40°C for further batch analysis to measure the 25(OH) vitamin D level in the preserved samples using a DRG 25OH vitamin D ELISA kit. Serum calcium, phosphate, alkaline phosphatase, and albumin were measured using the chemical autoanalyzer. Urine calcium and phosphorus were measured in a 24-hour urine sample. Patients were labeled as vitamin D deficient, insufficient, and sufficient if their levels were between <30 nmol/lit, 30–75 nmol/lit, and >75 nmol/lit, respectively. Statistical analysis was done using Microsoft Excel 2007 and SPSS version 16. Student's *t*-test and chi-square test were used with the level of significance set at ≤0.05.

## 3. Results

The average age of CAD patients was 54 years and that of the control group was 48 years (Table 1). 76% of the CAD patients were men, whereas in the control group, men comprised 42.5% of the subjects. Out of 121 CAD patients, 44% presented with the acute coronary syndrome and the remaining subjects had stable angina and ischemic cardiomyopathy. In the CAD group, 51.2% and 44.6% of the subjects had deficient and insufficient serum vitamin D levels, respectively (Table 2). Similarly, among 80 controls, 50% and 38.8% of the subjects had deficient and insufficient serum vitamin D levels, respectively. The clinical and biochemical characteristics of the cases and controls are shown in Table 1. The average duration of sun exposure (hours/day) among the CAD patients was higher than that among the controls (*P*=0.001). The mean waist-to-hip ratio, implying central obesity, was higher in CAD patients than that in the controls (*P*=0.04). Serum albumin (*P* < 0.0001) and corrected serum calcium levels (*P* < 0.0001) were lower in CAD patients than those in the controls. We did not find a significant difference in the levels of serum vitamin D (*P*=0.25), serum alkaline phosphatase (*P*=0.10), serum phosphate (0.06), 24-hour urine calcium (*P*=0.41), and phosphorus (*P*=0.30) between the CAD patients and controls.

## 4. Discussion

Vitamin D deficiency is prevalent in both CAD patients and healthy controls. Although CAD patients were slightly more deficient in vitamin D than the controls, the difference was not statistically significant. Our study reinforces the findings of previous studies [6–9] and suggests that vitamin D deficiency is widely prevalent in Indian population despite the presence of abundant sunshine. Interestingly, on analysis of the serum vitamin D level with respect to the length of time of sun exposure, the average time of sun exposure/day was higher in the CAD patients than that of the controls. However, increased sun exposure did not translate into increased serum vitamin D levels in CAD patients. Binkley et al. in their study supported our finding and proposed that this might be due to variations in individual response to ultraviolet B rays, which are mainly responsible for vitamin D synthesis [17]. It is also important to note the time of the day during which sun exposure occurs as maximum ultraviolet B rays exposure occurs during 11 am to 1 pm [18]. In addition to sun exposure, increased pigmentation of the Indian population may also affect cutaneous vitamin D synthesis [19].

We did not find any significant difference between the serum vitamin D levels in the CAD patients and controls. In a cross-sectional observational study, Dhibar et al. [12] concluded that vitamin D deficiency is also prevalent in subjects with angiography-proven normal coronary artery, and vitamin D deficiency and severity of deficiency does not correlate with angiographic severity of the disease. Karur et al. [13] in their study noted that vitamin D deficiency is widely prevalent in newly diagnosed CAD patients presenting with myocardial infarction. However, they did not include the control group for comparison; also, their study population included subjects with diabetes mellitus which itself has been shown to be associated with vitamin D deficiency and therefore can confound the association of vitamin D deficiency with CAD [20]. Similarly, Syal et al. [15] reported a higher prevalence of vitamin D deficiency in angiographically proven CAD patients and noted a positive association with severity of CAD and endothelial dysfunction with vitamin D deficiency, but their study lacks a control group. Shanker et al. [16] also reported an increased prevalence of vitamin D deficiency in CAD patients but did not exclude subjects with diabetes mellitus which is a confounding factor, therefore weakening the association. Interestingly, Rajasree et al. [14] in their study reported a higher prevalence of elevated vitamin D and calcium levels in CAD patients than controls and proposed the hypothesis of vitamin D-mediated arteriolar calcification leading to atherosclerosis.

In our study, the serum calcium level was lower in the CAD patients than that of the controls. Lu et al. [21] reported higher in-hospital mortality in patients admitted with ST-elevation myocardial infarction and a lower serum calcium level on admission in Chinese population. Rajasree et al. [14] reported a higher prevalence of above-normal calcium levels in patients with acute myocardial infarction than controls. Shiyovich et al. [22] proposed that serum calcium is an independent predictor of in-hospital mortality with U-shaped association implying both increased as well as decreased levels are associated with increased in-hospital mortality among patients admitted with acute myocardial infarction.

Our study had several limitations. First, because of the descriptive nature of the study and lack of randomization, observed association could be influenced by the unknown confounders and causality cannot be assessed. Second, small sample size and restriction to a single center limit the external validity of the study. Third, we did not have outcome-related data. Fourth, we did not have data related to the skin pigmentation and time of the day of sun exposure of the study subjects.

## 5. Conclusion

Vitamin D deficiency is widely prevalent in the Indian subcontinent despite the availability of abundant sunshine, and the level of serum vitamin D is not associated with the duration of sun exposure. Vitamin D deficiency is not associated with CAD. However, serum calcium is deficient in the CAD patients as compared to the controls. In the Indian population where vitamin D deficiency is widely prevalent and data on the normal serum vitamin D level are not available, the importance of a control group in future studies for comparison should be overemphasized. Given the lack of the studies exploring the association of vitamin D deficiency in CAD patients and conflicting results, large-scale multicentric prospective studies with controls are required to examine the association further and to evaluate the benefits of screening and correction of vitamin D deficiency in patients with CAD.

## Figures and Tables

**Table 1:** Clinical and biochemical characteristics of the cases and controls.

Variable	Cases *N* = 121		Controls *N* = 80		*P* values
	Mean	SD	Mean	SD	
Age (years)	54.22	9	48.9	6	0.001
Average sun exposure (hours/day)	3	2.1	1.88	1.27	0.001
BMI (kg/m^2^)	24.31	0.12	24.86	0.07	0.31
W/H ratio	0.98	0.12	0.94	0.07	0.04
Serum alkaline phosphatase (IU/L)	126.06	0.3	138.24	0.29	0.10
Serum albumin (g/dl)	4.21	5.3	4.75	4.31	<0.0001
Corrected serum calcium (mg/dl)	9.26	7	9.59	4.59	<0.0001
Serum phosphate (mg/dl)	3.80	38	4.02	35.9	0.06
Serum 25(OH)D (nmol/l)	34.06	18.79	40.19	27.8	0.25
24-hour urine calcium (mg/d)	1075.71	89.56	747.08	100.01	0.41
24-hour urine phosphorus (mg/d)	471.77	381.29	468.85	401.86	0.30

BMI: body mass index, W/H: waist-to-hip ratio, S 25(OH)D: serum vitamin D.

**Table 2:** Serum 25(OH)D in cases and controls.

25(OH)D level	Case	Control
<30 nmol/L (deficiency)	62 (51.2%)	40 (50%)
30 to 75 nmol/L (insufficiency)	54 (44.6%)	31 (38.8%)
>75 nmol/L (sufficiency)	5 (4.1%)	9 (11.2%)
Total	121	80

## Data Availability

The data used to support the findings of this study are available from the corresponding author upon request.
